# Zone-Shrinking Fresnel Zone Travel-Time Tomography for Sound Speed Reconstruction in Breast USCT

**DOI:** 10.3390/s20195563

**Published:** 2020-09-28

**Authors:** Xiaoyue Fang, Yun Wu, Junjie Song, Hang Yin, Liang Zhou, Qiude Zhang, Zhaohui Quan, Mingyue Ding, Ming Yuchi

**Affiliations:** Department of Biomedical Engineering, College of Life Science and Technology, Huazhong University of Science and Technology, Wuhan 430074, China; D201677469@hust.edu.cn (X.F.); D201980522@hust.edu.cn (Y.W.); junjiesong91@hust.edu.cn (J.S.); yinhang@hust.edu.cn (H.Y.); D201780498@hust.edu.cn (L.Z.); zhangqiude@gmail.com (Q.Z.); zh_quan@hust.edu.cn (Z.Q.); myding@hust.edu.cn (M.D.)

**Keywords:** USCT, sound speed, Fresnel zone tomography, breast cancer

## Abstract

Many studies have been carried out on ultrasound computed tomography (USCT) for its potential application in breast imaging. The sound speed (SS) image modality in USCT can help doctors diagnose the breast cancer, as the tumor usually has a higher sound speed than normal tissues. Travel time is commonly used to reconstruct SS image. Raypath travel-time tomography (RTT) assumes that the sound wave travels through a raypath. RTT is computationally efficient but with low contrast to noise ratio (CNR). Fresnel zone travel-time tomography (FZTT) is based on the assumption that the sound wave travels through an area called the Fresnel zone. FZTT can provide SS image with high CNR but low accuracy due to the wide Fresnel zone. Here, we propose a zone-shrinking Fresnel zone travel-time tomography (ZSFZTT), where a weighting factor is adopted to shrink the Fresnel zone during the inversion process. Numerical phantom and in vivo breast experiments were performed with ZSFZTT, FZTT, and RTT. In the numerical experiment, the reconstruction biases of size by ZSFZTT, FZTT, and RTT were 0.2%~8.3%, 2.3%~31.7%, and 1.8%~25%; the reconstruction biases of relative SS value by ZSFZTT, FZTT, and RTT were 24.7%~42%, 53%~60.8%, and 30.3%~47.8%; and the CNR by ZSFZTT, FZTT, and RTT were 67.7~96.6, 68.5~98, and 1.7~2.7. In the in vivo breast experiment, ZSFZTT provided the highest CNR of 8.6 compared to 8.1 by FZTT and 1.9 by RTT. ZSFZTT improved the reconstruction accuracy of size and the relative reconstruction accuracy of SS value compared to FZTT and RTT while maintaining a high CNR similar to that of FZTT.

## 1. Introduction

Ultrasound-computed tomography (USCT) systems capture both reflected and transmitted ultrasound signals [1,2,3,4]. The reflected signals are used to generate the reflection image, also known as the B-mode image of the hand-held ultrasound, while the transmitted signals are utilized to reconstruct the sound speed (SS) image and attenuation image [5,6,7,8]. The SS image and attenuation image can provide quantitative information [9,10] for diagnosis, e.g., a breast tumor normally has higher SS and attenuation coefficient than normal tissues [11,12,13,14]. One commonly used SS image reconstruction method is travel-time tomography [14,15,16,17] that adopts the information of travel time. Compared to waveform tomography [8,12,13] that adopts the information of waveform, travel-time tomography is more computationally efficient and more stable. Raypath travel-time tomography (RTT) assumes the wave propagates through a raypath based on the approximation of infinitely high wave frequency, which is sensitive to the noise in the detected travel time. Hence, the ability of RTT to identify an object from the background corrupted by noise (measured by contrast to noise ratio, CNR) is limited. Considering that the frequency is finite in ultrasound application, Fresnel zone travel-time tomography (FZTT) was presented under the assumption that the wave propagated not through a single ray but a zone called Fresnel zone [18,19,20,21]. Fresnel zone is an ellipsoidal region between a transmitter and a receiver, which can be interpreted as a region where the scattered waves interfere with the direct wave [18]. FZTT can provide the SS image with a high CNR, but the accuracy is low because the wide Fresnel zone smooths the image. Here, by introducing a weighting factor to shrink the Fresnel zone, we propose a zone-shrinking Fresnel zone travel-time tomography (ZSFZTT) to improve CNR and reconstruction accuracy.

In Section 2, the USCT system is introduced. Then, the concept of the Fresnel zone is explained. After that, the main ideas of ZSTT and ZSFZTT are described and illustrated by a simulated phantom model. Section 3 presents the experiments on a numerical breast phantom and in vivo breast. The reconstruction results by RTT, FZTT, and ZSFZTT are compared and analyzed. Section 4 gives the conclusions, limitations, and future directions of research.

## 2. Methods

### 2.1. USCT System

**Figure 1:**
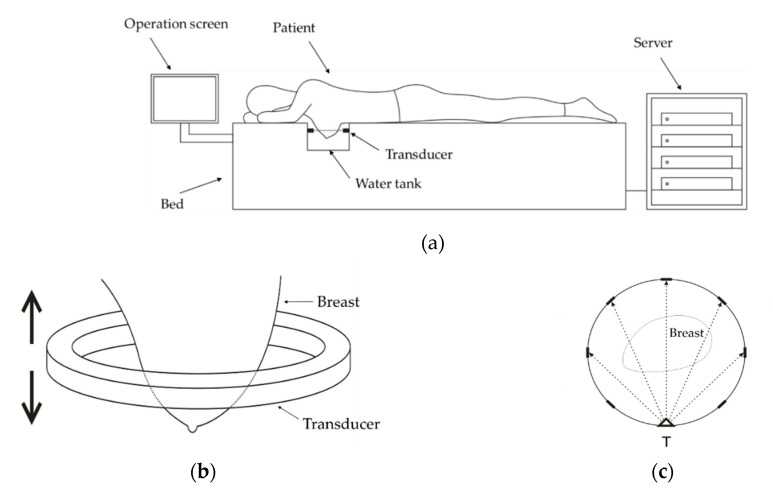
(**a**) The diagram of the ultrasound computed tomography (USCT) system; (**b**) the breast and the transducer; (**c**) the illustration of transmitting and receiving ultrasound signals.

In this research, the USCT system with a ring array transducer [22,23,24,25,26] is adopted. The diagram of the system is shown in Figure 1. In Figure 1a, the patient lies prone on the bed and puts one breast in the central area of the ring array transducer, which is immersed in the water. The operator performs the scanning and data acquisition on the operation screen. In Figure 1b, the transducer is moved vertically by a motor to capture the slice data of the coronal plane. The data is transferred to the server for image reconstruction. The ring array transducer is composed of thousands of transducer elements. In Figure 1c, the mode of transmitting and receiving signals is illustrated: when one element (represented by T) is activated to transmit the ultrasound signal, all the elements receive signals. The elements are activated one by one, until all the elements have transmitted signals. Assume that the number of transducer elements is $n_{E}$, then the amount of all the signals is ${n_{E}}^{2}$.

### 2.2. Fresnel Zone

**Figure 2:**
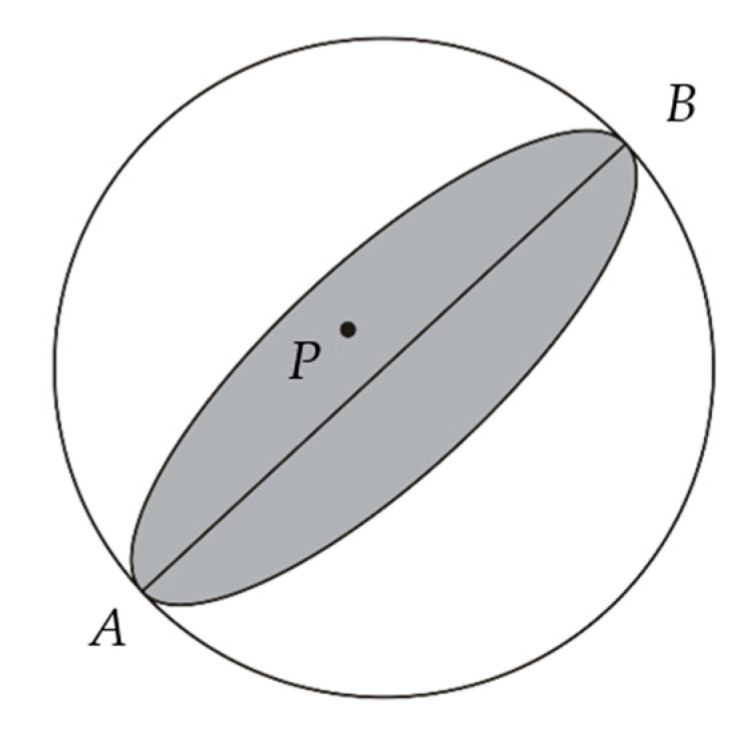
Illustration of the Fresnel zone.

The Fresnel zone is an ellipsoidal region between a transmitter and a receiver, which can be interpreted as a region where scattered waves interfere with the direct wave. Different from the raypath, the Fresnel zone has considered the scattering effect of the wave propagation. A simplified ring array transducer is shown in Figure 2 to illustrate the Fresnel zone. A and B are two of the transducer elements on the ring. A is the transmitter, B is the receiver, and P is an arbitrary spatial point. The straight line that links A and B is the central raypath. Considering that the frequency in USCT is finite, for the transmitter-receiver pair A  and B, the points that affect wave propagation are not only on the raypath but through a zone around the central ray, called the Fresnel zone [18,19,20,21]. In Figure 1, the gray part represents the Fresnel zone between A and B.

The Fresnel zone can be calculated from the eikonal equation

(1) $${\left(\frac{\partial T}{\partial x}\right)}^{2}+{\left(\frac{\partial T}{\partial y}\right)}^{2}=s^{2},$$

where T is the traveltime, x, y are two dimensional coordinates, and s is the slowness. By solving the eikonal equation with the finite difference (FD) method [27], the travel times among the spatial points in the imaging area can be obtained. The range of the Fresnel zone is determined under the condition [18]

(2) $$\Delta t=t_{AP}+t_{BP}-t_{AB},$$

(3) $$\Delta t_{max}=\frac{3}{8f},\;$$

(4) $$\Delta t<\Delta t_{max},$$

where Δt is the travel time delay between the detour path A→P→B and the direct path A→B, $t_{AP}$ is the travel time from transmitter A to point P, $t_{BP}$ is the travel time from receiver B to point P, $t_{AB}$ is the travel time from transmitter A to receiver B. $\Delta t_{max}$ is the constraint of travel time delay between the detour path and the direct path to determine the Fresnel zone, and f is the center frequency of the signal. The Fresnel zone between A and B illustrated by the gray area in Figure 2 is composed of the points that satisfy Equations (2)–(4).

### 2.3. Fresnel Zone Travel-Time Tomography (FZTT) and Zone-Shrinking FZTT (ZSFZTT)

**Figure 3:**
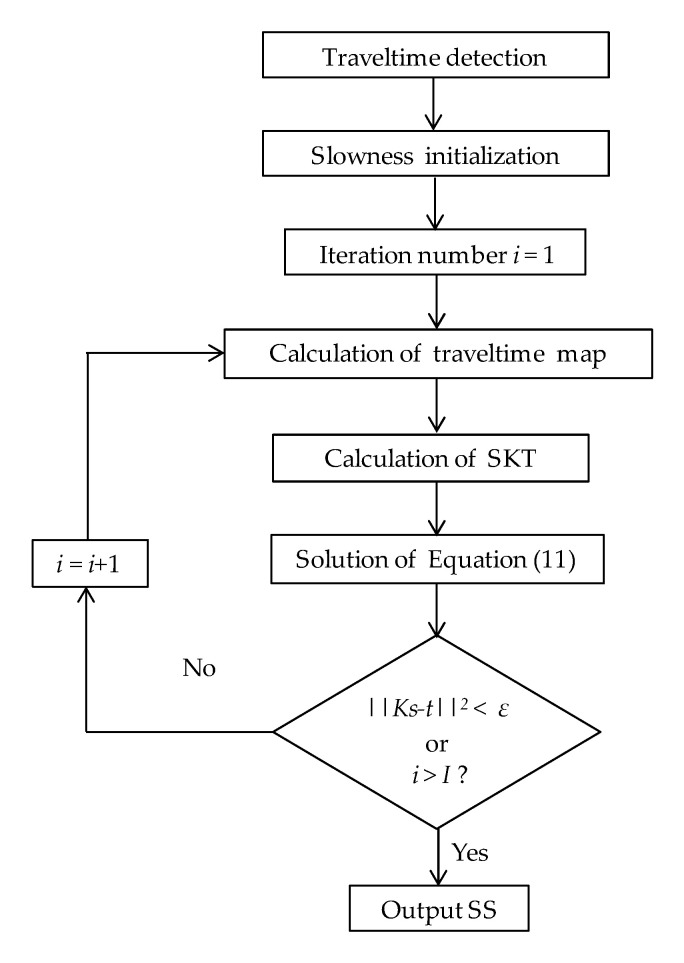
The flowchart of Fresnel zone travel-time tomography (FZTT) and zone-shrinking FZTT (ZSFZTT).

Fresnel zone travel-time tomography (FZTT) is an iterative inversion algorithm. The flowchart of FZTT algorithm is showed in Figure 3. Firstly the traveltime t is detected from the captured ultrasound signals by the Akaike information criterion (AIC) method [28]. Then set an initial slowness s and start the iterations. Calculate the traveltime maps for all transmitters by the FD method [27]. Δt is calculated by Equation (2) for all the transmitter-receiver pairs and to determine the Fresnel zone.

For transmitter-receiver A and B in Figure 2, in raypath travel-time tomography,

(5) $$t=\sum^{\;}L\left(P\right)s\left(P\right),$$

where t is the travel time from *A* to B, L(P) is the raypath on P, s(P) is the slowness (inverse of sound speed) on P. In Fresnel zone travel-time tomography,

(6) $$t=\sum^{\;}K\left(P\right)s\left(P\right),$$

where K(P) is the sensitivity kernel of travel time (SKT) [18], which reflects the sensitivity of t to the propagation medium. The higher the value of K(P), the more energy travels through P. After the Fresnel zone is determined, the SKT is approximated by [18]

(7a) $$\alpha =\{\begin{array}{ll} 1-\frac{8}{3}f\left|\Delta t\right|, & if\;0\leq \left|\Delta t\right|\leq \frac{3}{8f}\\ 0, & if\;\left|\Delta t\right|>\frac{3}{8f} \end{array},$$

(7b) K(P)=α∗g,

where α is a weighting parameter, K(P) is the SKT on point P, and g is the length of a grid cell.

**Figure 4:**
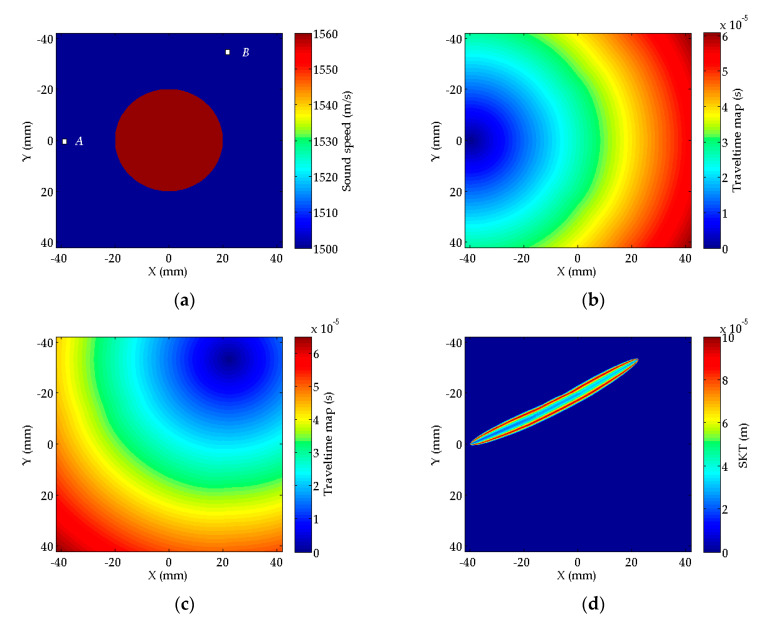
(**a**) A circular phantom immersed in water with a transmitter–receiver pair A−B; (**b**) travel-time map originated from transmitter A; (**c**) travel-time map originated from receiver B; (**d**) the SKT between transmitter A and receiver B calculated by Equation (7).

To illustrate the SKT in the Fresnel zone, a phantom model is simulated. Figure 4a shows the model: a circular phantom (red) with SS 1560 m/s is immersed in water (blue) with SS 1500 m/s. When the transmitter positioned at A is activated, the traveltime map originated from A obtained by the FD method [27] is plotted in Figure 4b. In the same way, when a receiver positioned at B is activated, the travel-time map originated from B can be obtained and plotted in Figure 4c. Using Equation (7), we can obtain the SKT in the imaging area (Figure 4d). The SKT appears as a “banana-doughnut” shape as indicated in Marquering’s [29] and Jocker’s [30], the values of SKT in the central area of Fresnel zone are smaller than those on the boundary of Fresnel zone. Here, the SKT appears slightly bent because of the refraction phenomenon.

After SKT for all transmitter–receiver pairs are calculated by Equation (7), FZTT is formulated as an optimization problem with an objective function F. Assuming the number of transmitter–receiver pairs is M, the imaging grids are of size N×N. The length of the square imaging area is D, which is the same as the diameter of the transducer. Then the grid size is

(8) g=D/N.

To prevent an underdetermined problem, we set

(9) N×N≤M.

Submitting Equations (8) to (9),

(10) $$g\geq D/\sqrt{M}.$$

where K is the SKT matrix of size $M\times N^{2}$, the slowness s is of size $N^{2}\times 1$, and the traveltime t is of size M×1. Then the objective function is designed as

(11) $$F=\underset{s}{\mathrm{argmin}}{\left|\left|Ks-t\right|\right|}^{2},$$

which can be solved by the Quasi-Newton methods. Here, a limited-memory Broyden–Fletcher–Goldfarb–Shanno (L-BFGS) method [31] from the family of Quasi-Newton methods is adopted. By solving Equation (11), the optimal value of s is obtained. Repeat the loop until the value of the objective function F is smaller than a predefined threshold ε determined by experience (approximately 200~500) or the iteration number i arrives at a predefined maximum iteration number I. Finally, the SS is formed by 1/s.

FZTT can reconstruct an SS image with a high CNR, but due to the wide Fresnel zone, the reconstructed SS image is usually inaccurate. Here, a zone-shrinking Fresnel zone travel-time tomography (ZSFZTT) is proposed to solve the problem. A weighting factor n is introduced to shrink the zone. Equation (12) shows how the weighting factor n is performed.

(12) $$\Delta t_{max}=\frac{3}{8nf},\;n=1,\;2,\;3,\ldots .$$

With the increase of n, $\Delta t_{max}$ decreases, which means fewer spatial points satisfy $\Delta t<\Delta t_{max}$; thus, the zone shrinks. In the proposed ZSFZTT, the calculation of the SKT is described by Equation (13),

(13a) $$\alpha =\{\begin{array}{ll} 1-\frac{8}{3}nf\left|\Delta t\right|, & if\;0\leq \left|\Delta t\right|\leq \frac{3}{8nf}\\ 0, & if\;\left|\Delta t\right|>\frac{3}{8nf} \end{array},$$

(13b) K(P)=α∗g.

The values of SKT get smaller because α decreases with the increase of n. Compared to FZTT, the calculation of Fresnel zone uses Equation (12) instead of Equation (3), and the calculation of SKT uses Equation (13) instead of Equation (7).

**Figure 5:**
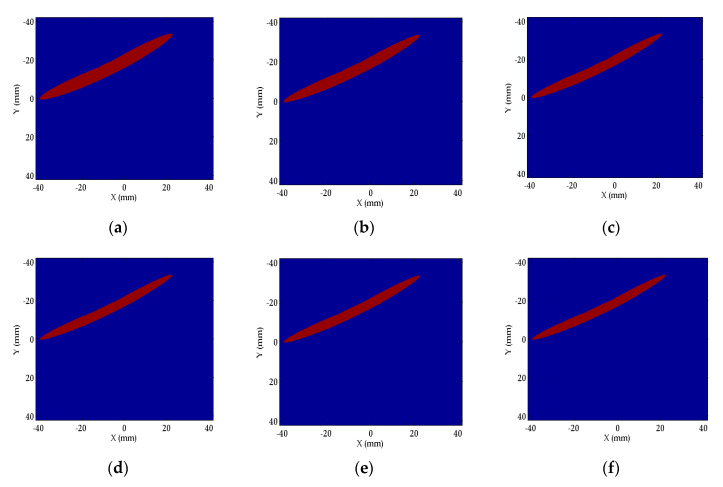
The Fresnel zone regulated by n between transmitter A and receiver B when (**a**) n=1; (**b**) n=2; (**c**) n=3; (**d**) n=4; (**e**) n=5; (**f**) n=8.

We tested how the Fresnel zone shrinks when n increases. The Fresnel zone between transmitter A and receiver B when n=1; 2; 3; 4; 5; 8 is plotted in red in Figure 5. We can find that the Fresnel zone shrinks when n=1→2→3→4, while the zone does not shrink significantly when n>4. Compared to the zone area when n=1, the zones’ areas when n=2;3;4;5;6;7;8 shrink to 85.7%, 80.3%, 77.5%, 75.7%, 74.5%, 73.7%, and 73.0% respectively. When n>4, the decrease of the percentage with the increase of n is less than 2%. n this research, n is imposed with a constraint defined by Equation (14) during the inversion process,

(14) $$n=\{\begin{array}{cc} i, & if\;1\leq i\leq 4\\ 4, & \;if\;i>4 \end{array},$$

where i is the iteration number.

## 3. Experiments and Results

To evaluate the ZSFZTT algorithm, a numerical experiment and an in vivo experiment were conducted. The numerical data were simulated by the open source acoustic toolbox k-wave [32]. The SS image was reconstructed by ZSFZTT and compared to RTT and FZTT. The in vivo experiment used the data of a patient who was diagnosed with invasive breast cancer. The data were captured by the USCT system [23,24,25,26] developed in the Huazhong University of Science and Technology, China. The slice containing the lesion was reconstructed by ZSFZTT and compared to RTT and FZTT.

### 3.1. Quantitative Evaluation Metrics

The SS image is evaluated quantitatively by four metrics defined by Equations (15)–(18), below. The reconstruction bias is calculated for both size and SS value to evaluate reconstruction accuracy. The lower the bias, the higher the accuracy is. The reconstruction bias of size ($Bias_{size}$) is defined as

(15) $$Bias_{size}=\frac{\left|\bar{D}-D_{d}\right|}{D_{d}}\times 100\%,$$

where $\bar{D}$ is the average measured diameter of the object or the background from the reconstructed SS image, $D_{d}$ is the designed diameter of the object or the background. The reconstruction bias of SS ($Bias_{SS}$) is defined as

(16) $$Bias_{SS}=\frac{\left|\bar{SS}-SS_{d}\right|}{SS_{d}}\times 100\%,$$

where $\bar{SS}$ is the average measured SS value of the object or the background from the reconstructed SS image, $SS_{d}$ is the designed SS value of the object or the background. To evaluate the relative reconstruction accuracy of SS value, the relative reconstruction bias of SS ($Relative\_Bias_{SS}$) is defined as

(17) $$Relative\_Bias_{SS}=\left(1-\frac{\left|{\bar{SS}}_{o}-{\bar{SS}}_{b}\right|}{\left|SS_{od}-SS_{bd}\right|}\right)\times 100\%,$$

where ${\bar{SS}}_{o}$ is the average measured SS value of the object, ${\bar{SS}}_{b}$ is the average measured SS value of the background, $SS_{od}$ is the designed SS value of the object, $SS_{bd}$ is the designed SS value of the background. The lower the $Relative\_Bias_{SS}$, the higher the relative reconstruction accuracy of SS value.

The ability to detect mass or tumor from the background that contains noise is measured by the contrast to noise ratio (CNR) [33], defined by

(18) $$CNR=\frac{\left|{\bar{SS}}_{o}-{\bar{SS}}_{b}\right|}{\sigma_{b}},$$

where $\sigma_{b}$ is the standard deviation of the background’s SS value. The higher the CNR, the more easily the object can be identified.

### 3.2. Numerical Breast Phantom Experiment

**Figure 6:**
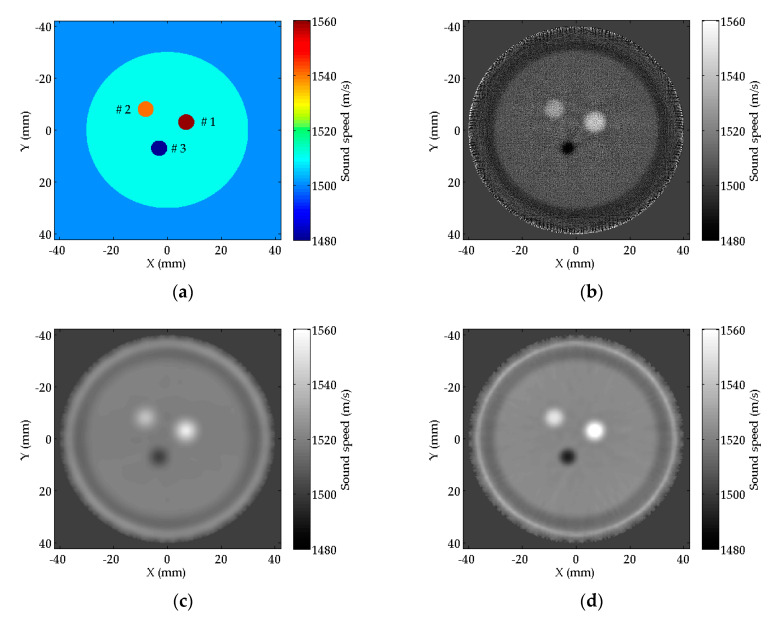
(**a**) The designed numerical breast phantom model; (**b**) reconstructed sound speed (SS) image of the numerical breast phantom by raypath travel-time tomography (RTT); (**c**) reconstructed SS image of the numerical breast phantom by FZTT; (**d**) reconstructed SS image of the numerical breast phantom by ZSFZTT.

The open source acoustic toolbox k-wave in MATLAB [26] was used to generate simulation data. Figure 6a shows the setup of the numerical experiment: a circular breast phantom with three circular masses inside. The background of the phantom is set to mimic the normal breast tissue. The Mass #1 and Mass #2 are set to mimic the tumors with higher SS values than normal breast tissue. The Mass #3 is set to mimic the cyst with a lower SS value than normal breast tissue. Table 1 gives the size and SS values of the breast phantom. The breast phantom is scanned by a ring array transducer immersed in water. The number of the transducer elements is 512, the center frequency of the transducer is 3.0 MHz. The diameter of the ring array transducer, the phantom and the three masses are 80 mm, 60 mm, and 6 mm, respectively. The SS value of water is 1500 m/s. In the data generation, the wavelength is 0.5 mm, and five sampling points per wavelength are set to satisfy the requirement of k-wave toolbox that at least three sampling points per wavelength. When one transducer element transmits a signal, all the transducer elements receive signals. The number of the numerical signals is 512^2^.

In the inversion of the numerical breast phantom data, we use 385 receiver elements opposite to the transmitter. These elements cover about 270° of the ring array, and are considered to receive the transmission signals, while the remaining elements receive reflection signals. Thus, the amount of the transmitter-receiver pairs is M=512×385. The diameter of the transducer is 80 mm, which means D=80 mm. We set g=0.2 mm to prevent the underdetermined problem.

The SS images reconstructed and by RTT, FZTT, and ZSFZTT are plotted in Figure 6b–d. Figure 6b shows the SS image reconstructed by RTT. The circular shape of the three masses can be distinguished from the background. Mass #1 and #2 are brighter than the background, while Mass #3 is darker than the background, which corresponds to the designed contrast of SS values. Mass #3 in Figure 6b is smaller than the designed size. Figure 6c shows the SS image reconstructed by FZTT, the three masses are also visible but are with low contrast compared to the background. Figure 6d shows the SS image reconstructed by ZSFZTT, the three masses are clearly distinguished with high contrast from the background and the size of the three masses is closer to the designed size.

**Figure 7:**
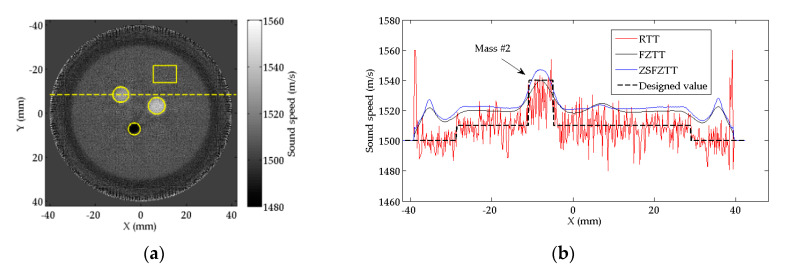
(**a**) The horizontal central line (the dashed line) to be measured in Mass #2 and area of interest (AOI) selection (the circles and the rectangle) for measurements; (**b**) the profiles of SS values on the horizontal central line of Mass #2.

To compare the image details, the profiles of SS value on the central line (illustrated by the dashed line in Figure 7a) of the Mass #2 are plotted in Figure 7b. We can find that the profiles of FZTT and ZSFZTT are smooth and uniform, while the profile of RTT is fluctuant. The recognition of Mass #2 (where the solid arrow points out) is clear in FZTT and ZSFZTT. There is an overall rise on the SS value by FZTT and ZSFZTT, which is possibly caused by the approximation scheme of the SKT defined by Equations (7) and (13).

To quantitatively evaluate the reconstruction accuracy, the reconstruction biases of RTT, FZTT, and ZSFZTT are summarized in Table 2, Table 3 and Table 4. Note that the diameter of the background represents the diameter of the phantom. The lower the bias, the more accurate the reconstruction is. The lowest bias is marked by bold characters. All the measurements are repeated and averaged in manually selected areas of interest (AOIs, the circles for the masses and the rectangle for the background, in Figure 7a). From Table 2, we can find that ZSFZTT has the lowest $Bias_{size}$ for all the three masses which means that ZSFZTT can enhance the reconstruction accuracy of size. From Table 3, we can find that ZSFZTT has lowest $Bias_{SS}$ for Mass #1 and Mass #2. However, for the background and Mass #3, $Bias_{SS}$ by ZSFZTT is higher compared to RTT. In ZSFZTT, the inconsistent result about $Bias_{SS}$ is from the overall rise on the SS value that is shown in Figure 7b. Furthermore, we compared relative reconstruction accuracy of SS value which is evaluated by $Relative\_Bias_{SS}$ summarized in Table 4. We can find that ZSFZTT has the lowest $Relative\_Bias_{SS}$ for all the three masses, which indicates that ZSFZTT can enhance the relative reconstruction accuracy of SS value.

To quantify the ability to detect the masses from the background, CNR of the three masses are measured on the same AOI mentioned above. We can find from Table 5 that FZTT and ZSFZTT have significantly higher CNR than RTT, which is mainly because of the decline of the standard derivation of the background. FZTT has the highest CNR for Mass #1, Mass #2. ZSFZTT has the highest CNR for Mass #3 even though the standard derivation of the background by ZSFZTT is higher than FZTT. In general, ZSFZTT maintains the CNR that is comparable to that of FZTT.

It should be noted that, since AOI is manually selected based on the brightness of the gray level, although multiple measurements are taken to give average value, the measurement of the reconstructed size still includes measurement bias. Moreover, since the sound speed value in the AOI is not uniform, select a larger AOI will result in a decrease in the average value. Although measurement bias exists, the above measurements are convincing for experimental comparison because the same AOI selection criteria are used.

### 3.3. In Vivo Breast Experiment

To further evaluate the proposed ZSFZTT, an in vivo breast experiment was conducted using the USCT system [23,24,25,26] developed in the Huazhong University of Science and Technology, China. The procedures for the patient experiment were approved by the Ethics Committee of the Tongji Medical College, Huazhong University of Science and Technology. The USCT system uses a ring array transducer with 2048 elements; the diameter of the transducer is 220 mm; the center frequency is 3.0 MHz. A 40-year-old female patient was scanned, in whose right breast an invasive breast cancer was diagnosed by pathological examination. In the inversion of the in vivo data, we used 1537 receiver elements opposite to the transmitter (following the same rule in the numerical experiment, i.e., the range of 270° of the ring array). Thus, the amount of the transmitter–receiver pairs is M=2048×1537. The diameter of the transducer is 220 mm, which means D=220 mm. We set g=0.8 mm to prevent the underdetermined problem.

**Figure 8:**
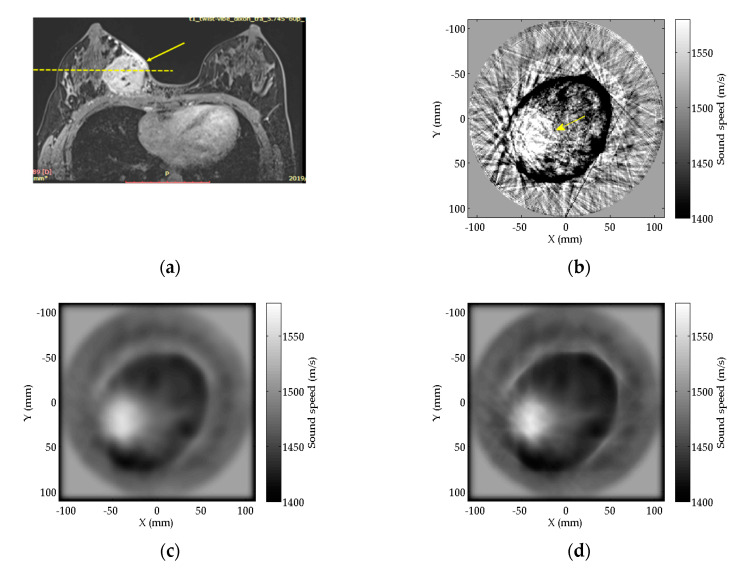
(**a**) MRI result of the patient’s breast; (**b**) reconstructed SS images of the patient’s breast by RTT; (**c**) reconstructed SS images of the patient’s breast by FZTT; (**d**) reconstructed SS images of the patient’s breast by ZSFZTT.

The magnetic resonance Imaging (MRI) result of the breast with invasive cancer in the transverse plane is showed in Figure 8a for reference, in which the arrow points to the tumor. The SS images of the slice (approximately illustrated by the dashed line in Figure 8a) where the tumor located are reconstructed by RTT, FZTT, and ZSFZTT (Figure 8b–d). As the breast is immersed in water during the scan process of USCT, the shape of the breast in the SS image differs from that in the MRI image. The bright mass pointed out by the arrow in the left bottom of the breast in Figure 8b is the tumor. We can find that the RTT gives the result with artefacts, while FZTT and ZSFZTT provide smoother results with less artefacts in Figure 8c,d.

**Figure 9:**
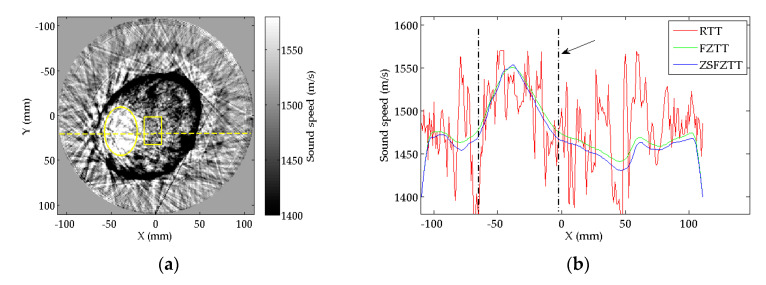
(**a**) The horizontal central line (the dashed line) to be measured in tumor and AOI selection (the circle and the rectangle) for measurements; (**b**) the profiles of SS value on the horizontal central line of the tumor.

The profiles of SS value on the central line (the dashed line in Figure 9a) of the tumor is plotted in Figure 9b; the dash-dotted lines outline the tumor area from the normal tissues. In RTT, there are fluctuant noises that may obscure the recognition of the tumor, of which SS value varies from 1400 m/s to 1600 m/s. Similar to the results of numerical phantom experiment, the profiles of FZTT and ZSFZTT are smoother than that of RTT.

The CNR of the three methods are summarized in Table 6. The AOI is illustrated in Figure 9a, the circle indicates the tumor and the rectangle indicates the normal tissue, i.e., the background. The CNR of ZSFZTT is 8.6, higher than 1.9 for RTT and 8.1 for FZTT. The measurements of CNR indicate that the ability to detect the tumor is enhanced by ZSFZTT. As there is no true SS value of the tumor, the reconstruction accuracy is not evaluated.

## 4. Conclusions

Sound speed is a quantitative parameter measured in breast USCT. In this work, sound speed reconstruction using travel-time tomography in breast USCT is studied. RTT and FZTT are two methods of the family of travel-time tomography. The image reconstructed by RTT has low CNR. FZTT can provide image with high CNR but low accuracy. This work proposed ZSFZTT to enhance both CNR and accuracy. By a numerical breast phantom experiment and an in vivo breast experiment, ZSFZTT was evaluated and compared to RTT and FZTT. In the numerical breast phantom experiment, compared to RTT and FZTT, ZSFZTT improved the reconstruction accuracy of size and the relative reconstruction accuracy of SS value; ZSFZTT maintained high CNR that was comparable to that of FZTT. In the in vivo breast experiment, ZSFZTT provided the highest CNR compared to RTT and FZTT. The experiments showed that ZSFZTT can enhance the reconstruction accuracy and maintain a high CNR. The reason that FZTT and ZSFZTT resulted in lower standard deviations and higher CNR is probably because the assumption of wave propagation through Fresnel zone has smoothing effect, which need be explored further. There still remain some limitations and challenges of this study. In the zone-shrinking process of ZSFZTT, the weighting factor n was roughly designed, and the physical significance of the zone-shrinking strategy requires deeper understanding. Additionally, there was an overall rise on the sounds speed value reconstructed by FZTT and ZSFZTT, which may be caused by the approximation of the SKT. In the future, more experiments should be conducted to verify the efficiency of ZSFZTT. Deeper investigation on wave propagation through Fresnel zone is needed. Moreover, some regularization techniques can be introduced to the reconstruction process to improve the image quality.

## Figures and Tables

**Table 1:** The design of the numerical breast phantom.

	Background	Mass #1	Mass #2	Mass #3
$SS_{d}$ (m/s)	1510	1560	1540	1480
$D_{d}$ (mm)	60	6	6	6

**Table 2:** The $Bias_{size}$ measured on SS image of numerical phantom reconstructed by RTT, FZTT, and ZSFZTT methods.

Methods	Diameter (mm)				
		Background	Mass #1	Mass #2	Mass #3
	$D_{d}$	60	6	6	6
RTT	$\bar{D}$	61.1	7.5	6.8	4.8
	$Bias_{size}$	1.8%	25%	13.3%	20%
FZTT	$\bar{D}$	61.4	7.9	7.6	6.6
	$Bias_{size}$	2.3%	31.7%	26.7%	10%
ZSFZTT	$\bar{D}$	60.1	6.2	6.1	5.5
	$Bias_{size}$	**0.2%**	**3.3%**	**1.7%**	**8.3%**

Note: Bold characters mark the lowest bias.

**Table 3:** The $Bias_{SS}$ measured on SS image of numerical phantom reconstructed by RTT, FZTT, and ZSFZTT methods.

Methods	Sound Speed (m/s)				
		Background	Mass #1	Mass #2	Mass #3
	$SS_{d}$	1510	1560	1540	1480
RTT	$\bar{SS}$	1507.0	1533.1	1523.7	1486.1
	$Bias_{SS}$	**0.2%**	1.7%	1.1%	**0.4%**
FZTT	$\bar{SS}$	1520.5	1540.1	1534.2	1506.4
	$Bias_{SS}$	0.7%	1.3%	0.4%	5.4%
ZSFZTT	$\bar{SS}$	1522.8	1551.8	1543.1	1500.2
	$Bias_{SS}$	0.8%	**0.5%**	**0.2%**	1.4%

Note: Bold characters mark the lowest bias.

**Table 4:** The $Relative\_Bias_{SS}\;$ measured on SS image of numerical phantom reconstructed by RTT, FZTT, and ZSFZTT methods.

Methods		Background	Mass #1	Mass #2	Mass #3
RTT	$\bar{SS}$ (m/s)	1507.0	1533.1	1523.7	1486.1
	$Relative\_Bias_{SS}$	--	47.8%	44.3%	30.3%
FZTT	$\bar{SS}$ (m/s)	1520.5	1540.1	1534.2	1506.4
	$Relative\_Bias_{SS}$	--	60.8%	54.3%	53%
ZSFZTT	$\bar{SS}$ (m/s)	1522.8	1551.8	1543.1	1500.2
	$Relative\_Bias_{SS}$	--	**42%**	**33.3%**	**24.7%**

Note: bold characters mark the lowest $Relative\_Bias_{SS}$. “--” represents no value.

**Table 5:** The CNR  measured on SS image of numerical phantom reconstructed by RTT, FZTT, and ZSFZTT methods.

Methods		Background	Mass #1	Mass #2	Mass #3
RTT	$\bar{SS}\pm \sigma$ (m/s)	1507.0 ± 9.6	1533.1 ± 11.7	1523.7 ± 12.0	1486.1 ± 6.5
	CNR	--	2.7	1.7	2.2
FZTT	$\bar{SS}\pm \sigma$ (m/s)	1520.5 ± 0.2	1540.1 ± 6.7	1534.2 ± 2.9	1506.4 ± 2.9
	CNR	--	**98**	**68.5**	70.5
ZSFZTT	$\bar{SS}\pm \sigma$ (m/s)	1522.8 ± 0.3	1551.8 ± 7.9	1543.1 ± 5.0	1500.2 ± 5.2
	CNR	--	96.6	67.7	**75.3**

Note: Bold characters mark the highest CNR. “--” represents no value.

**Table 6:** The CNR  measured in SS image of the patient’s breast reconstructed by the RTT, FZTT, and ZSFZTT methods.

Methods		Normal Tissue	Tumor
RTT	$\bar{SS}\pm \sigma$ (m/s)	1463.9 ± 37.3	1534.7 ± 39.6
	CNR	--	1.9
FZTT	$\bar{SS}\pm \sigma$ (m/s)	1468.8 ± 6.6	1522.4 ± 11.7
	CNR	--	8.1
ZSFZTT	$\bar{SS}\pm \sigma$ (m/s)	1467.2 ± 7.2	1529.4 ± 12.1
	CNR	--	**8.6**

Note: bold characters mark the highest CNR. “--” represents no value.
